# Comparison of a fast track protocol and standard care after hip arthroplasty in the reduction of the length of stay and the early weight-bearing resumption: study protocol for a randomized controlled trial

**DOI:** 10.1186/s13063-021-05314-5

**Published:** 2021-05-17

**Authors:** Martina Rocchi, Cesare Stagni, Marco Govoni, Alessandro Mazzotta, Leonardo Vivarelli, Antonella Orlandi Magli, Mariada Perrone, Maria Grazia Benedetti, Dante Dallari

**Affiliations:** 1 Reconstructive Orthopaedic Surgery and Innovative Techniques - Musculoskeletal Tissue Bank, IRCCS Istituto Ortopedico Rizzoli, Via G.C. Pupilli 1, 40136 Bologna, Italy; 2 Physical Medicine and Rehabilitation Unit, IRCCS Istituto Ortopedico Rizzoli, Via G.C. Pupilli 1, 40136 Bologna, Italy; 3 Anesthesia, Intensive Care and Pain Therapy, IRCCS Istituto Ortopedico Rizzoli, Via G.C. Pupilli 1, 40136 Bologna, Italy

**Keywords:** Early mobilization, Education, Fast track, Hip arthroplasty, Pain management

## Abstract

**Background:**

To date, hip arthroplasty is one of the most commonly performed surgical procedures, with growing worldwide demand. In recent decades, major progress made in terms of surgical technique, biomechanics, and tribology knowledge has contributed to improve the medical and functional management of the patient. This study aims to assess if the application of a fast track protocol, consisting of a preoperative educational intervention, adequate postoperative pain control, and intensive rehabilitation intervention, reduces the length of stay (LOS) and allows the early functional recovery compared to standard clinical practice for patients undergoing hip arthroplasty.

**Methods:**

The study population consists of 90 patients with primary arthrosis of the hip with an anterior indication of hip arthroplasty. The exclusion criteria are older than 70 years, a contraindication to performing spinal anesthesia, and bone mass index (BMI) greater than 32. Participants, 45 for each group, are randomly allocated to one of two arms: fast track clinical pathway or standard care protocol. During allocation, baseline parameters such as Harris Hip Score (HHS) and Western Ontario and McMaster Universities (WOMAC) index are collected. On the third postoperative day, the functional autonomy for each patient is assessed by the Iowa Level of Assistance (ILOA) scale, and it is expected the discharge for patients in the fast track group (primary outcome). On the other hand, standard care patient discharge is expected after 5–7 days after surgery. During follow-up fixed at 6 weeks and 3, 6, and 12 months, HHS and WOMAC scores are collected for each patient (secondary outcomes).

**Discussion:**

Although total hip replacement has become a widespread standardized procedure, to the authors’ knowledge, only few randomized controlled trials were performed to evaluate the effectiveness of fast track pathway vs. standard care procedure in the reduction of the LOS after hip arthroplasty. It is expected that our results collected by the application of minimally invasive surgical interventions with concomitant management of perioperative pain and bleeding and early functional rehabilitation will contribute to enriching the understanding of clinical and organizational aspects linked to fast track arthroplasty.

**Trial registration:**

ClinicalTrials.gov NCT03875976. Registered on 15 March 2019—“retrospectively registered”.

## Background

Total hip replacement (THR) is one of the most successful surgeries, with more than 1 million procedures undertaken annually worldwide [1]. THR has become established as an elective method of treating end-stage arthritis of the hip with excellent long-term outcomes [2]. The average aging of the population, the increase in life expectancy, the arthrosis conditions secondary to sports injuries or any other source of physical trauma are the basis for the increasingly growing demand for arthroplasty [3, 4].

On the other hand, over the last decades, there have been many advances in the fields of biomechanics, tribology, prosthetic design, and surgical technique [5, 6] that have contributed to minimize the patient discomfort after arthroplasty favoring the reduction of the hospital recovery [7]. Moreover, in a climate of limited resources and global financial strain, ways of containing costs and pressure from third-party payers, maintaining at the same time highly efficient care pathways, better patient satisfaction, and improved patient-reported outcomes are needed [2, 8].

Therefore, at present, the attention of the scientific community is focused on the quality of the result achieved and the subjective experience of the patient [9]. In particular, the rapid recovery during the postoperative stage has become a central element, and it is considered as a synthesis of the most advanced surgical and medical practices [10, 11]. The concept of the multimodal approach to the surgical patient was introduced in the 1990s by Professor Henrik Kehlet [12]. He developed the concept of “Fast Track,” a methodology that focuses on optimizing clinical outcomes in synergy with logistical improvements to achieve a quick admission of the patient and a reduction of his length of stay (LOS) and convalescence. This procedure, also known as enhanced recovery after surgery (ERAS), is possible through adequate perioperative assistance, efficient use of available resources, and considering the patient as a central and active role in the rehabilitation process. Therefore, to date, total hip arthroplasty (THA) and total knee arthroplasty (TKA) are established procedures for the effective treatment of complications of advanced arthritis that have contributed to decreasing the average length of stay in hospital.

Recently, there is a growing interest in performing joint arthroplasty on an outpatient or short-stay basis. However, in order to perform a successful outpatient arthroplasty program, a robust screening of patients to ensure the selection of appropriate candidates is needed. Thus, in comparison with patients who underwent to fast track protocol, the patients recruited for outpatient joint arthroplasty are younger and more active and have suffered from less medical comorbidities than the more typical lower limb arthroplasty patients. As a consequence, fast track protocol is still primarily performed [13].

Regarding hip arthroplasty, although fast track protocol in THR has become a well-defined trend and many data have been available since its development [14–16], many difficulties for widespread implementation of these findings should be addressed in order to adjust the fast track pathway based on scientific evidence. Also, although randomized controlled trials are considered a powerful tool in evidence-based medicine for evaluating the effects of medical interventions, in our knowledge, only few studies were conducted by a rigorous way to determine the cause-effect relation existing between the fast track and standard care procedures [17–19].

Thus, in this study, we propose a randomized controlled hip fast track protocol to confirm the safety and feasibility of the procedure and precluding eventual perioperative complications in the clinical pathway applied. Besides, this trial aimed to establish what drawback or shortcoming may arise to a 3-day discharge to limit or avoid adverse effects and ameliorate short-term clinical outcomes.

## Methods/design

### Objectives

The main purpose of the controlled intervention trial is to evaluate if the application of a fast track protocol, compared to the standard clinical practice, can allow early functional recovery after hip replacement surgery. In detail, the objectives of this intervention are as follows: (a) improving the clinical-functional outcome of the hip surgical intervention, (b) minimizing the impact of the procedure on the patient’s quality of life, and (c) reducing the economic-social costs that the standard clinical treatment path involves.

### Outcome measures

Outcomes will be collected at different time points during a total of 12 months, and the primary endpoint is at 3 days after a hip surgery.

#### Primary outcome

##### Iowa Level of Assistance (ILOA) scale

The primary outcome is the evaluation of the early clinical-functional improvements assessed by the ILOA scale at the third postoperative day. This scale is a 6-item and 36-point tool used to value reliability, validity, and responsiveness of functional tests in patients with total joint replacement [20]. The total score can vary from 0 to 50, where 50 indicates the higher disability.

#### Secondary outcomes

##### Harris Hip Score (HHS)

HHS is a clinician-based outcome measure developed to evaluate various hip disabilities such as pain, function, absence of deformity, and range of motion in an adult population [21]. Each item has a unique numerical scale, which corresponds to descriptive response options and scores range from 0 to 100 (higher scores representing less dysfunction and better outcomes). HHS is collected at allocation and during follow-up fixed at 6 weeks and 3, 6, and 12 months.

##### Western Ontario and McMaster Universities (WOMAC) index

WOMAC is a self-administered questionnaire that probes the health status of patients with lower extremity osteoarthritis [22]. It consists of 24 items to assess pain, stiffness, and physical function with 5, 2, and 17 questions, respectively. Each question is rated on an ordinal scale of 0 to 4, with lower scores indicating lower levels of symptoms or physical disability. WOMAC index is collected at allocation and during follow-up fixed at 6 weeks and 3, 6, and 12 months.

##### Numeric Rating Scale (NRS)

NRS is a pain scale in which the patient indicates their subjective pain as a whole number from 0 to 10 [23]. The participant is asked to report their hip pain and discomfort using NRS, where 10 indicates “the most severe pain and discomfort imaginable.” Patients are asked to rate their pain by NRS on the day of surgery and in the following days of recovery in the hospital.

##### Postoperative blood management

Postoperative blood transfusions are common in total hip arthroplasty because of preoperative anemia and perioperative blood loss. Although both fast track and standard care protocols apply the same procedures to control erythropoiesis and bleeding, the number of postoperative blood transfusions is collected in the case report form (CRF).

##### Postoperative pain relief

Scheduled postoperative pain therapy and the potential administration of rescue doses during analgesic therapy are collected in the CRF of each treatment arm.

##### Length of stay (LOS)

As reported in the work of Mota [24], the LOS for primary THR ranged between 5 and 11 days in five general Italian hospitals. In our Institute, as reported in the SPIRIT chart (Fig. 2), the LOS for patients with age ≤ 70 years, BMI < 32, and ASA ≤ 2 ranged between 5 and 7 days. Thus, it is expected that performing fast track protocol on the same selected population reduces the LOS between 2 and 4 days.

##### Analysis of costs

The treatment costs incurred by the clinical trial will be calculated considering the center’s costs data per day of admission for each group. Since the main economic difference between the fast track and standard care protocols is based on the LOS, cost savings will be calculated as the difference between the mean stay for each treatment arm multiplied for corresponding daily cost.

##### Adverse events

Intra- and postoperative adverse events (AEs), such as intraoperative complications, re-admission, and postoperative complication rates, will be recorded in the CRF and addressed by medical operators according to our standard clinical guidelines.

Potential AEs will also be collected during each outpatient visit by a medical operator not involved in the conduct of the study.

In addition, in order to establish the safety, study conduct, and scientific validity and integrity of the trial, each reported AE is evaluated by an independent clinical trial monitor by an audit performed every 4 months. Thus, the clinical monitor will provide recommendations to the principal investigator as to whether the study should continue without change, be modified, or be terminated.

### Protocol and study design

**Fig. 1:**
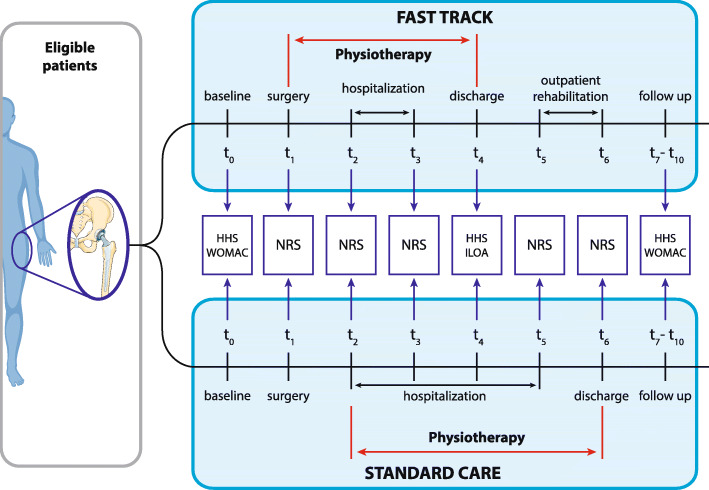
Scheme of the study design. *HHS* Harris Hip Score, *ILOA* Iowa Level of Assistance, *NRS* Numeric Rating Scale, *WOMAC* Western Ontario and McMaster Universities

**Fig. 2:**
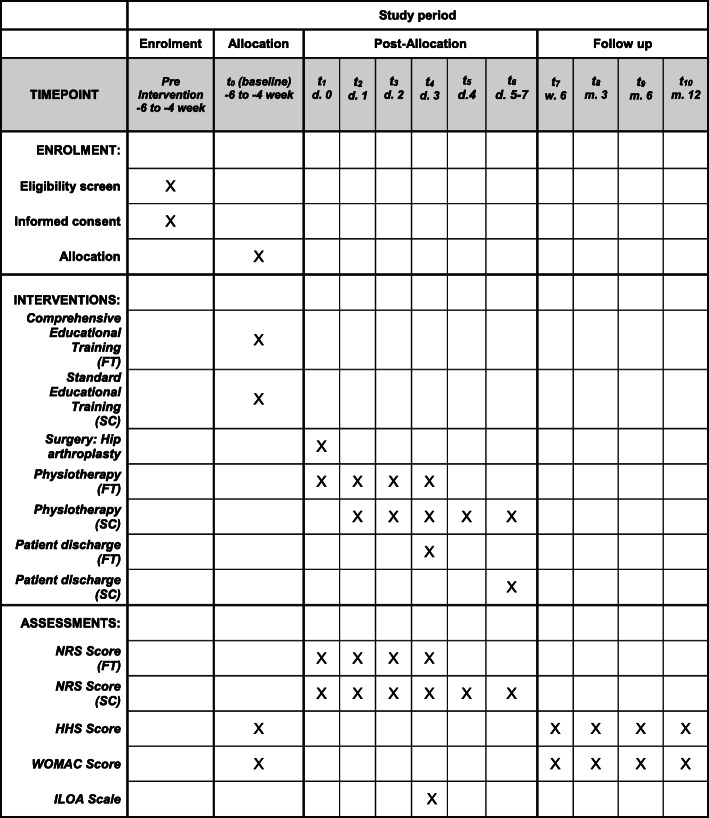
Schedule of enrollment, intervention, and assessments in the study. *FT* fast track, *SC* standard care, *d* day, *w* week, *m* month

An overview of the study is reported in Table 1, Fig. 1, and the SPIRIT chart (Fig. 2). This intervention trial is an open-label controlled randomized trial of arthroplasty for primary osteoarthritis of the hip designed to include at least 90 patients, 45 for each treatment arm.

**Table 1:** Summary scheme of fast track vs. standard care interventions

Operative stage	Fast track	Standard care
Preoperative	*Comprehensive patient education* - 1-h lesson - Multimedia material (images, animation, and video) - Patient personal summary booklet	*Standard patient education* - 15-min lesson
Intraoperative	- Minimally invasive surgery - Subarachnoid anesthesia - Tranexamic acid	- Minimally invasive surgery - Subarachnoid anesthesia - Tranexamic acid
Pain management	*2 h before surgery:* - 1 tablet of 1000 mg paracetamol - 1 tablet of 600 mg gabapentin - 1 tablet of 200 mg celecoxib	
	*4 h after surgery:* - 1 tablet of 1000 mg paracetamol - 1 tablet of 300 mg gabapentin *Before sleeping:* - 1 tablet of 10 mg oxycodone or 1 tablet of 100 mg tapentadol *The first postoperative day:* - 1 tablet of 1000 mg paracetamol every 6 h - 1 tablet of 30 mg celecoxib at 8.00 a.m. - 1 tablet of 300 mg gabapentin at 9.00 a.m. *From the second postoperative day up to a maximum of 15 days:* - 1 tablet of 1000 mg paracetamol every 12 h - 1 tablet of 200 mg celecoxib at 8.00 a.m.	*Every 8 h after surgery* - 30 mg ketorolac and 100 mg tramadol in 100 mL of physiologic saline solution *Severe pain management* - 1 tablet of oxycodone hydrochloride (5 or 10 mg) is administered every 12 h
	*(Rescue dose):* - 1 tablet of 500 mg paracetamol - 1 tablet of 30 mg codeine phosphate (this dose should be repeated after 12 h)	*(Rescue dose):* - Intravenous 1000 mg paracetamol every 8 h
Rehabilitation	*Intensive rehabilitation protocol* Day 0 (t_1_): - In the morning: surgical operation Day 0 (t_1_), in the afternoon: - Resuming verticalization - Walking with the help of physiotherapists and a front-wheel walker	*Standard rehabilitation protocol* Day 0 (t_1_): - In the morning: surgical operation
	Day 1 (t_2_): - Mobilization in bed - Attempt to use crutches - Stair climbing with crutches (if tolerated) - Physiotherapy is performed two times a day	Day 1 (t_2_), in the morning: - Mobilization in bed Day 1 (t_2_), in the afternoon: - Resuming the vertical position - Walking with the help of physiotherapists and a front-wheel walker
	Day 2 (t_3_): - Verification and control of functional acquisitions of the patient - Weaning from a crutch - Physiotherapy twice daily	Day 2 (t_3_): - Physiotherapy is performed twice a day using first the walker then the crutches
	Day 3 (t_4_): - Weight-bearing gradual resumption - Patient discharge	Days 3, 4 (t_4_, t_5_): - Stair climbing with crutches - Weaning from a crutch
		Days 5–7 (t_6_): - Weight-bearing gradual resumption - Patient discharge

The study population consists of patients affected by primary hip arthrosis with the indication for hip arthroplasty performing by anterior approach. Recruited patients are randomly assigned to the fast track (FT) or standard care (SC) group. In case of admission to the study, an informed consent form must be signed by the participant before participating in one of the two procedures provided in the intervention.

The course of the study is explained to each potential participant during the pre-admission visit (t_0_), planned and coordinated by an experienced medical team. Each patient is subjected to blood tests for the evaluation of hemochrome and indices of nonspecific phlogosis and x-rays of the pelvis and affected hip. A specific patient teaching is scheduled only for patients assigned to the FT group, as exhaustively reported in the “Interventions” section.

Both groups are subjected to a minimally invasive surgery performed with erythropoiesis and bleeding control (t_1_), whereas only FT patients are asked to follow a short-term antalgic therapy and a 3-day intensive rehabilitation protocol that begins about 4 h after surgical intervention.

In order to reduce bias between groups related to the problem of different skill levels of surgeons, surgical interventions in FT and SC patients are performed by the same expert surgeon of the Reconstructive Orthopaedic Surgery and Innovative Techniques Unit. Besides, regarding hip prostheses, in this study protocol it is planned to use the same models of short uncemented stems, hemispherical acetabular cups, and ceramic-ceramic coupling for both treatment arms, with the aim of avoiding postoperative differences related to the features of the devices. However, during surgical interventions, it may happen that some patients need a standard stem. Thus, information regarding the use of different stems is collected on the CRF and considered during pain management and data analysis, although recent meta-analyses of randomized clinical trials show that both types of stems achieve similar radiological and clinical outcomes [25, 26].

On the day of surgery (t_1_) and in the following days of hospitalization, patients are asked to rate their pain by NRS. Pain scores are collected on CRF, and a NRS ≤ 3 is defined as the value for considering patients fit for discharge.

Three days after surgical intervention (t_4_), the Iowa Level of Assistance Scale (ILOA), a valid and reliable measure for patients following THR, as reported in several studies [27–30], is collected to measure the early functional autonomy of patients—i.e., primary outcome. Among the indication for THR, there are particularly pain and impaired physical function, which are the two dominating domains in HHS [31, 32]. On the other hand, the WOMAC index, being a self-administered questionnaire validated for osteoarthritis in the lower extremities, is usually used as a disease-specific instrument to assess the quality of life for patients with THR [33, 34]. Therefore, in this study protocol, the data from HHS and WOMAC are collected during allocation to reach baseline parameters (t_0_) for each participant. These scores are also collected by outpatient appointments during follow-up fixed at 6 weeks and 3, 6, and 12 months (t_7_, t_8_, t_9_, and t_10_, respectively) to assess the improvements of patient health status (secondary outcomes). All data are collected by medical outcome assessors not involved in surgical interventions.

Compared to the use of questionnaires drafted via telephone or mail, in our experience, outpatient appointments favor more continued participation from the outset. In addition, in order to promote patient retention to the study, some strategies such as creating a welcoming environment, establishing an efficient tracking system of patients, and educating participants about their role as research participants are used. The request for further rehabilitation after discharge in the two groups will also be monitored. As reported in the informed consent, each participant has the right to withdraw from any aspect of the trial at any time. Nevertheless, patients will continue to receive the appropriate therapeutic treatment to address their primary hip osteoarthritis in case of withdrawal.

The study is conducted following the ISO 14155:2011 and Good Clinical Practice (GCP) for the design, conduct, recording, and reporting of clinical investigations carried out in human subjects.

The Ethics Committee of the Rizzoli Orthopaedic Institute (Bologna, Italy) approved the trial on 12 January 2018. Then, the Ethics Committee of Area Vasta Emilia Centro - Regione Emilia-Romagna (CE-AVEC) approved a protocol amendment that contained a modification to the pain management protocol on 12 September 2018 (*EM432/2018_87/2017/Sper/IOR_EM1*). Specifically, since etoricoxib was no longer available in the hospital pharmacy, it was replaced with celecoxib, a drug essentially similar in terms of the level of pain relief.

### Participants

Patients referred for this trial are screened for eligibility according to the principle of inclusion and exclusion criteria, as reported in Table 2. The study population consists of patients with primary osteoarthritis of the hip with the indication of anterior hip arthroplasty. As reported by Pulido et al. [35], although total joint arthroplasty is a safe surgical procedure, it may be associated with rare serious and life-threatening complications. In particular, advanced age and severe comorbidities are identified as significant risk factors, which also caused a considerable variation in the LOS with reported stays of 5–11 days [3, 36].

**Table 2:** Principle of inclusion and exclusion criteria

Inclusion criteria	Exclusion criteria
Male or female between the age of 18 and 70 years	Lack of written consent
ASA score ≤ 2	Patients with cognitive impairments and psychiatric diseases
BMI < 32	ASA ≥ 3
	Each stage of RA
Preoperative hemoglobin level > 13 g/dL	Preoperative use of crutches
Caregiver presence	Contraindications for the use of spinal anesthesia
	No caregiver presence

Therefore, although an unselected population provides a more useful assessment of effectiveness, a homogeneous study population makes the evaluation of efficacy more straightforward, especially for a small sample size population. Consequently, the majority of THR studies have employed a selected population with restrictions on age, BMI, and the severity of comorbidities [37].

According to these studies, patients older than 70 years with a BMI greater than 32 are here excluded. Besides, the American Society of Anesthesiologists (ASA) physical health status classification [38] is used as inclusion/exclusion criteria. Specifically, patients with some functional limitation due to diseases, such as diabetes, chronic renal failure, heart and respiratory diseases, and implanted pacemaker, are excluded (ASA ≥ 3). In addition, while poor preoperative status determined by the ASA scores are recognized as risk factors for short-term complications after THA and TKA [39, 40], the risk contributed by rheumatoid arthritis (RA) has not been as well defined [41]. Therefore, as reported in Table 2, each stage RA patient is excluded.

In the Emilia Romagna region (Italy), the last Regional Register of Orthopaedic Prosthetic Implantology (RIPO) report [42], referred to 2017, shows that about 53% of patients who underwent primary THR is younger than 70 years.

For this protocol study, also considering BMI < 32 and ASA ≤ 2 as inclusion criteria, a 10% reduction of eligible patient number is expected.

Patients are recruited at the time of clinical-instrumental assessment as required by the usual clinical practice during the 4–6 weeks before admission.

After the patients’ consent to inclusion in the trial, they are screened at the Reconstructive Orthopaedic Surgery and Innovative Techniques Unit of the IRCCS Istituto Ortopedico Rizzoli (Bologna, Italy), and baseline medical history, including general medical history, is recorded. In detail, a baseline measure of WOMAC and HHS scores is collected with an interview about quality of life and medication usage. The baseline assessment is collected on the day of the pre-admission visit (t_0_). The absence of functional autonomy of the patient—i.e., the preoperative use of crutches—is considered as an exclusion criterion.

### Interventions

#### Fast track protocol

##### Comprehensive preoperative patient education

Preoperative teaching is considered an essential part of patient care since it can prevent complications and promote patient fulfillment during LOS.

Preoperative patient education is performed on the day of pre-admission visit (t_0_): it consists of 1-h lesson for a maximum of five patients accompanied by a relative. This lesson begins with the patient interview to obtain comprehensive information about the patient. It continues with a detailed description of the surgical protocol, the surgical access, the type of prosthesis, and the anesthesiological procedure used during the intervention. Moreover, the physiotherapy exercises are well explained with particular regard to the description of the roles of physiotherapists, anesthetists, and surgeons, who work together in order to provide effective patient-centered care. Multimedia material, such as images, animation, and video, is also showed to facilitate patient comprehension. Finally, patients are provided with a personal summary booklet.

##### Erythropoiesis and bleeding control

The intravenous infusion of 10 mg/kg of tranexamic acid (TXA) is scheduled at the induction of anesthesia, 10 mg/kg at the early stage of the surgical procedure, and an additional dose of 10 mg/kg after 6 h leaving the operating room. Surgical drainage is not required.

##### Perioperative pain management

The intervention is conducted under subarachnoid anesthesia by 12 mg of levobupivacaine and without the Foley catheter. Moreover, a perioperative multimodal antalgic treatment followed by a standardized scheme of oral therapy (gabapentin and an NSAID with a rescue dose in the case of NRS ≥ 3) is performed.

In detail, the perioperative pain management is as follows:

i. 2 h before surgery: 1 tablet of 1000 mg paracetamol; 1 tablet of 600 mg gabapentin; 1 tablet of 200 mg celecoxib.
ii. 4 h after surgery: 1 tablet of 1000 mg paracetamol; 1 tablet of 300 mg gabapentin.
iii. *Rescue dose:* 1 tablet of 500 mg paracetamol; 1 tablet of 30 mg codeine phosphate (this dose should be repeated after 12 h).
iv. Before sleeping: 1 tablet of 10 mg oxycodone or 1 tablet of 100 mg tapentadol. Tapentadol is administered only in case the patient is already treated by this drug during the preoperative stage for the treatment of chronic pain.
v. The first postoperative day: 1 tablet of 1000 mg paracetamol every 6 h, 1 tablet of 30 mg celecoxib at 8.00 a.m., and 1 tablet of gabapentin 300 mg at 9.00 a.m.
vi. *Rescue dose:* 1 tablet of 500 mg paracetamol and 1 tablet of 30 mg codeine phosphate (this dose should be repeated after 12 h).
vii. From the second postoperative day up to a maximum of 15 days: 1 tablet of 1000 mg paracetamol every 12 h and 1 tablet of 200 mg celecoxib at 8.00 a.m.

##### Rehabilitation protocol

In detail, the rehabilitation protocol is as follows:

i. Day 0 (t_1_), in the morning: surgical operation.
ii. Day 0 (t_1_), in the afternoon: resuming verticalization; walking with the help of physiotherapists and a front-wheel walker.
iii. Day 1 (t_2_): mobilization in bed; attempt to use crutches; stair climbing with crutches (if tolerated).
iv. Physiotherapy is performed two times a day.
v. Day 2 (t_3_): verification and control of functional acquisitions of the patient; weaning from a crutch.
vi. Physiotherapy is needed two times a day.
vii. Day 3 (t_4_): weight-bearing gradual resumption; patient discharge.

#### Standard care protocol

##### Preoperative patient education

A standard patient education is scheduled on the day of pre-admission visit (t_0_): it consists of a visit with the orthopedic surgeon for the collection of informed consents and with the anesthesiologist. The average duration of the medical meeting is about 15 min. Patients are not provided with a personal summary booklet.

##### Erythropoiesis and bleeding control

Both FT and SC protocols apply the same procedures to control erythropoiesis and bleeding.

##### Perioperative pain management

The intervention is conducted under subarachnoid anesthesia by 12 mg of levobupivacaine and without the Foley catheter. Pain management follows the standard procedure of the IRCCS Istituto Ortopedico Rizzoli: the patients receive 30 mg ketorolac and 100 mg tramadol in 100 mL of physiologic saline solution, 8 h after surgery. These dosages are repeated every 8 h, and in case of severe pain, one tablet of oxycodone hydrochloride (5–10 mg) is administered every 12 h.

*Rescue dose:* intravenous 1000 mg paracetamol every 8 h.

##### Rehabilitation protocol

In detail, the rehabilitation protocol is as follows:

i. Day 0 (t_1_), in the morning: surgical operation.
ii. Day 1 (t_2_), in the morning: mobilization in bed.
iii. Day 1 (t_2_), in the afternoon: resuming the vertical position; walking with the help of physiotherapists and a front-wheel walker.
iv. Day 2 (t_3_): physiotherapy is performed twice a day using first the walker then the crutches concerning the degree of confidence with the assistive devices.
v. Days 3, 4 (t_4_, t_5_): stair climbing with crutches; weaning from a crutch.
vi. Days 5 – 7 (t_6_): weight-bearing gradual resumption; patient discharge.

### Sample size calculation, randomization, and statistical analysis

The sample size was calculated based on the primary outcome—i.e., score changes in the ILOA scale collected at the third postoperative day. Specifically, considering a standard deviation of the ILOA scale of ± 6.9, a difference of seven points deemed clinically significant [20, 29], and a power of 90% with a significance level of 0.05, the minimum number of patients to be enrolled is estimated at 40 per arm (1:1 ratio) [43]. Presuming a 10% drop-out rate, following potential rare severe complications during surgery, it is necessary to enroll 90 patients, 45 per arm.

The subjects are randomized to either the SC group or the FT group by the ER software [44] using the permuted blocks method [45] to randomly allocate the participants to each group in order to avoid imbalance in the number of participant assignment. Randomization, 1:1 ratio for each treatment arm, is performed centrally at the IRCCS Istituto Ortopedico Rizzoli by a colleague who is not involved in subject enrolment and is blinded to the participants, investigators/health care providers, or persons assessing outcomes. The randomization list is inserted in envelopes sequentially numbered and sealed; the investigator opens the envelopes in sequence on the day of enrolment.

A modified intention-to-treat (mITT) analysis is used to handle data of patients. Specifically, every subject who is randomized to each treatment group, excepted for patients with severe intraoperative complications, ignoring withdrawal, and anything that happens after surgery, is accounted for in the interpretation of results.

Patients are monitored at third postoperative day, at discharge, and during follow-up fixed at 6 weeks and 3, 6, and 12 months (according to standard clinical practice).

ILOA, NRS, WOMAC and HHS scores, LOS, the number of postoperative blood transfusions, the need for rescue doses during analgesic therapy and AEs are assessed to compare the two treatment arms. Outcome assessors and analysts are blinded.

Analyses are performed using GraphPad Prism ver. 6. For the values for which the mean and standard deviation is determined, a 95% confidence coefficient (CI 95%) is also determined, indicating a range of values with a 95% confidence level of a similar group mean. The ILOA difference between groups is calculated using the chi-square criterion method or Fisher’s direct test. A significant difference between groups is considered for *P* < 0.05. In addition, for the evaluation of secondary outcomes (i.e., scores for the NRS, WOMAC, HHS, LOS, the number of blood transfusion and rescue doses, AEs), a comparison between groups will be performed using the Student *t*-test if the data will be normally distributed. If not, the Mann-Whitney *U* test will be used to compare groups at each measurement time. Differences between groups are considered significant for *P* < 0.05. In case of data collected by primary and secondary outcome measurements will be affected by covariates (e.g., age, gender, BMI, ASA, short/standard stem, or the number of blood transfusions), a post hoc regression analysis will be performed to reduce any bias in the estimation of treatment effect which may occur by a potential random imbalance between groups [46].

### Confidentiality of data

Personal identity and all personal medical information of the patients are confidential. Each participant is assigned a unique ID number. The study participants’ consent is obtained before the trial.

## Discussion

Fast track surgery has evolved over the past 20 years and has proven its efficacy in terms of reducing hospital stay, morbidity, and convalescence without an increase in re-admission or safety rates [47]. In order to reduce risk factors that could increase perioperative complications or the chance of a new hospitalization within 3 months, multidisciplinary aspects related to the management of organizational aspects and patient education must be accurately addressed before the surgery. Thus, the introduction of the hip fast track protocol in our Institute has the purpose of improving (i) the THR procedure performance, (ii) time from admission to surgery, and (iii) length of stay.

To this aim, preoperative patient education, erythropoiesis and bleeding control, perioperative pain management, and rehabilitation protocol optimization are the main issues to address.

Yoon et al. [48] have already shown that adequate preoperative information reduces the LOS. Therefore, in our study, during the pre-admission visit, a detailed multidisciplinary interview with an anesthesiologist, a surgeon, and other professional figures such as physiatrists or psychologists proposes to provide exhaustive information on anesthesia, surgical techniques, and analgesics to minimize the degree of anxiety related to surgery and reducing pain during and after the intervention.

Furthermore, since in the scientific literature it is widely demonstrated that the presence of chronic preoperative pain is linked to a greater probability of developing or maintaining pain in the postoperative period [49–51], common risk factors (e.g., smoking, obesity, type 2 diabetes mellitus, and anemia) must be carefully assessed before the surgery.

THR can be burdened by significant blood loss and often requires transfusions: exposed bone surfaces, surgical trauma to tissues and blood vessels, fibrinolysis, and platelet dysfunction are just some complications that contribute to intraoperative bleeding. To reduce the total loss of blood, drugs with antifibrinolytic properties may be used (e.g., aprotinin, tranexamic acid, and epsilon-aminocaproic acid) [52]. Among these compounds, tranexamic acid (TXA) is a synthetic lysine analogue commonly used in many types of surgery, thanks to its ability to inhibit fibrinolysis and clot degradation [53]. Large randomized clinical trials and meta-analyses have consistently confirmed that the intravenous administration of TXA could effectively and safely reduce perioperative blood loss and transfusion, albeit it is not risk-free [54, 55]. If nausea, diarrhea, blurred vision, and headache are the most common side effects, a possible risk of thromboembolic events is the most severe complication. To avoid it, clinicians should strive to administer the minimal dose known to achieve their outcomes.

Nevertheless, a single-dose regimen of TXA does not give effective results. On the other hand, if a two-dose regimen is the least amount necessary for effective results, a three-dose regimen produced a maximum effective reduction of drain loss and total blood loss [56]. Larger doses do not provide additional hemostatic benefit. In accordance with Maniar et al*.* [56] and with our THR surgical experience, a three-dose regimen of TXA has shown to be the most efficacious, safe, and cost-effective dose to reduce bleeding.

Generally, THR is associated with significant perioperative pain, which can adversely affect recovery by increasing the risk of complications, length of stay, and costs. Inadequate pain management following THR may increase morbidity and mortality, decrease patient satisfaction, and lead to chronic persistent postsurgical pain [57]. Consequently, the current recommendations to pain management, also applied in our FT protocol, emphasize a multimodal approach based on the maximization of the positive aspects of the treatment, limiting the associated side effects. Since the opioid-related analgesic therapy causes many of negative side effects, limiting perioperative opioid use is a major principle of multimodal analgesia.

Despite their side effects, opioid analgesics continue to represent a cornerstone in postoperative pain control [58]. In our study protocol, SC patients are treated by intravenous loading of tramadol, a centrally acting analgesic that is structurally related to morphine and codeine, with low incidence of adverse effects such as respiratory depression, constipation, and abuse potential.

However, in order to reduce the incidence of nausea and other side effects and complication of opioids [59], FT patients are treated by gabapentin, a third-generation antiepileptic drug that also has demonstrated efficacy in treating neuropathic pain related to chronic pain under spinal anesthesia [60], and by a single dose of oxycodone that is well-tolerated via oral intake. Codeine, in association with paracetamol, is administrated exclusively as a rescue dose.

Since the pain management of FT patients is based on an oral drug administration, NSAIDs such as ketorolac used in SC protocol are replaced with celecoxib, a selective type 2 cyclooxygenase (COX-2) inhibitor, in order to limit gastrointestinal side effects such as ulcers or bleeding.

Besides, during the intraoperative phase, the choice of subarachnoid anesthesia instead of general anesthesia is driven by results of several studies that prove favorable outcome effects such as a reduction of endocrine metabolic response, a lower postoperative vomiting, and morphine consumption [61, 62].

Finally, since postoperative physiotherapy is an integral part of mobilization, intensive rehabilitative protocols are proposed for empowering the patient to regain independence as quickly as possible following surgery [63–65]. The rehabilitation program in the two groups is different substantially in time of recovery of walking and stair climbing with weight-bearing. In the FT protocol, the patient is usually operated in the morning and then in the afternoon subjected to an early-stage verticalization, starting to walk the same day of surgery and in the first postoperative day climbing stairs with partial weight-bearing with the help of physiotherapists and assistive devices.

After that, physiotherapy is performed twice a day for the following 2 days. On the third day, if the program is respected, the patient is discharged without crutches reaching a full weight-bearing resumption.

In the SC protocol, (i) walking is allowed in the first postoperative day with partial weight-bearing, (ii) stair climbing is allowed in the third or fourth postoperative day, and (iii) full weight-bearing after 5 or 7 days.

In addition, activities of daily life such as dressing and hygiene are resumed before discharge on the third postoperative day in FT patients. In contrast, in the SC protocol, these activities are carried out at the end of the hospital stay, generally after a week from surgical intervention.

After discharge, patients in both paths are invited to continue home exercises for the increasing hip range of motion, muscular strengthening, and proprioceptive recovery, but further rehabilitation sessions are not required. However, some patients may want to continue rehabilitation voluntarily. A recent study [66] has highlighted that, in a choice-based service model of “therapy as required” following hip and knee arthroplasty, only a third of THA and half of TKA patients accessed post-discharge therapy and patients who did not access physiotherapy reported greater postoperative outcomes. The request for further rehabilitation in both groups could bias the results of the present study, and therefore, the rehabilitation needs of FT and SC patients will be monitored in order to prevent potential crossover effect between groups that may occur due to the home rehabilitation period.

It is important to highlight that although one of the risks of early discharge from hospital is a higher frequency of re-admission, fast-track THR surgery does not increase the re-admission rate compared with conventional surgical pathways, as reported by Husted et al. [67]. Nevertheless, all major intraoperative and postoperative complications, which can potentially cause re-admissions, will be recorded to compare treatment arms.

In conclusion, while several hospitals of different countries have already adopted well-defined patient care pathways in hip fractures [68–72], in our knowledge, this is the first randomized controlled trial that compares fast track protocol and standard care in the reduction of the LOS and the early weight-bearing resumption in only 3 days after hip arthroplasty. Results collected by the application of minimally invasive surgical methods, management of perioperative pain and bleeding, and early functional rehabilitation will provide relevant new information on clinical and organizational aspects of fast-track THR, and detailed documentation of safety and patient satisfaction.

## Trial status

Recruitment started on 13 March 2018 using protocol version 1 released on 18 December 2017. The study is concluded in March 2021.

## Data Availability

The datasets generated and/or analyzed during the current study will be available from the lead author upon reasonable request.

## Abbreviations

AEs: Adverse events
ASA: American Society of Anesthesiologists
BMI: Body mass index
CRF: Case report form
FT: Fast track
GCP: Good Clinical Practice
HHS: Harris Hip Score
ILOA: Iowa Level of Assistance
ISO: International Organization for Standardization
LOS: Length of stay
NRS: Numeric Rating Scale
NSAIDs: Non-steroidal anti-inflammatory drugs
SC: Standard care
THA: Total hip arthroplasty
TKA: Total knee arthroplasty
THR: Total hip replacement
TXA: Tranexamic acid
WOMAC: Western Ontario and McMaster Universities
